# Dark Tourism in Southern Spain (Córdoba): An Analysis of the Demand

**DOI:** 10.3390/ijerph18052740

**Published:** 2021-03-08

**Authors:** María Genoveva Dancausa Millán, María Genoveva Millán Vázquez de la Torre, Ricardo Hernández Rojas

**Affiliations:** 1 Management, Economic Applied and Stadistics, Universidad de Córdoba, 14071 Córdoba, Spain; 2 Quantitative Methods, Universidad Loyola Andalucía, 14004 Córdoba, Spain; gmillan@uloyola.es; 3 Agricultural Economics, Finance and Accounting, Universidad de Córdoba, 14071 Córdoba, Spain; ricardo.hernandez@uco.es

**Keywords:** dark tourism, thanatourism, ghost tourism, ARIMA models

## Abstract

In recent decades, there has been a change in tourists’ tastes; they want to experience something novel. To satisfy this demand, a new type of tourism, known as “dark tourism”, has arisen; it has various modalities, among which cemetery tourism and ghost tourism stand out, in addition to very different motivations from those of the cultural tourist. In this type of tourism, cemeteries are not visited to appreciate their architecture or heritage but to explore a morbid curiosity about the people buried there; ghost tourism or paranormal tourism seizes on the desire to know the events that occurred there and tends to have macabre content. This study analyzes dark tourism in the province of Córdoba in southern Spain with the aim of knowing the profile of the tourist and his motivation. This study additionally will forecast the demand for this type of tourism, using autoregressive integrated moving average (ARIMA) models, which allow us to know this market’s evolution and whether any promotional action should be carried out to promote it.

## 1. Introduction

In recent years, tourists’ tastes have been changing in an observable way; they not only want to know the experiences of other people but are also looking for new and personal experiences. Therefore, tourism has adapted to these changing tastes [1]. The application of new technologies provides a consumer with an extensive range of tourism modes at the touch of a button as well as the opinions and ratings of previous consumers. These reviews can provide a degree of confidence about the target destination, especially those with few visitors due to divisive themes or macabre or spiritual connotations. There is newly emerging tourism demand related to places where a tragedy has happened [2,3,4,5].

Death, beyond the feeling of loss of a loved one, can be explored when the consumer has some emotional distance and the circumstances that have preceded death were abnormal; such things include tragedies from which we must learn so as not to repeat them. Clear examples of this include genocide [6] and slavery [7].

Since ancient times, some people have enjoyed and been fascinated with the trappings of death; this can be considered the forebearer of death tourism, minus the tourism because this occurred at home and was a leisure activity. Thus, in the era of the gladiators, Romans observed how these gladiators fought for their lives against other gladiators or beasts as a form of entertainment. In the Middle Ages, many people came to witness executions (deaths at the stake or hanging) as a fact of everyday life [8].

Most dark tourism researchers acknowledge that people traveled to places associated with death before the modern era. Seaton [9] linked such practices to a thanatotic tradition, which was a part of Western religious and philosophical.

However, it was during the 20th century that people begin to travel to visit places related to death but without the philosophical and spiritual connotation. These people visited rather from the point of view of curiosity or morbid curiosity; in the contemporary historical period (after the First World War), this became the origins of dark tourism, and its motivations are varied according to various death-related subsegments. In this work, we will analyze a subsegment of dark tourism and cemetery tourism in southern Spain. 

The main objective of this article is to provide knowledge in the field of dark tourism. Specifically, this study analyzes dark tourism in the province of Córdoba in southern Spain with the aim of knowing the profile of the tourist and his motivation. This study additionally will forecast the demand for this type of tourism, using autoregressive integrated moving average (ARIMA) models. This research deepens in this field which is not very studied, for this purpose first perform an academic definition and classification, then defines the so-called cemetery tourism and shows the results of a survey of this type of tourist in a specific case in the south of Spain. End discussions and conclusions. 

## 2. Dark Tourism and Classification

The tourism of death and suffering has had several names, such as morbid tourism [10], thanatourism [11], negative tourism [12], tragic tourism [13], grief tourism [14], milking the macabre [15], difficult heritage [16], atrocity tourism [17] or dark tourism [18], the latter being the most widely used coinage today. For these researchers, “dark tourism” is the travel of visitors to places where deaths or disasters have occurred. They do not participate to commemorate family and friends but as leisure or recreation, marking a fundamental change in the conception of how death, disaster and other atrocities are handled as associated tourist products. However, there are other definitions of this term, among which the Institute for Dark Tourism Research, University of Central Lancashire (England) stands out. Dark tourism is the act of travel and visitation to sites, attractions, and exhibitions that have real or recreated death, suffering or seemingly macabre as a main theme. Tourist visits to former battlefields, slavery-heritage attractions, prisons, cemeteries, particular museum exhibitions, Holocaust sites, or to disaster locations all constitute the broad realm of ”Dark Tourism” [19].

Biran et al., [20] indicates that this tourism has become a popular and emotive term that perhaps excessively simplifies a complex, multifaceted and multidimensional phenomenon; however, Lennon and Foley [18] indicate that dark tourism is the product of the circumstances of the modern world, where there is a change in the patterns of presentation and consumption of death in tourist destinations, which can vary depending on the place. According to Slade [21], tourists from Australia and New Zealand who visit Gallipoli do so not so much for being a place where there was a battle that resulted in many deaths but rather see it as the birth of their nations; the motive is national identity rather than death.

Dark tourism is increasingly in demand, but what drives people to do it? The reasons can be diverse, including the following:

Desiring experiences or to have “holidays in hell” [22].

Needing to look death in the face, or having a mere interest in death and everything that relates it [23].

Having an interest both in history and in the heritage it has left behind or in education and memories of the past and the suffering it has caused [24]. This interest is combined with the desire to understand how people can survive catastrophes and the attempt to pay homage to the people who suffered, learning a lesson from the past so as not to repeat it [25].

Conserving both the heritage of a place and its history and therefore the identity of a collective [26].

Satisfying our curiosity [27].

Being challenged or changing the perception of mortality [28,29].

Stone [30] created a framework for classifying different types of “dark tourism”. Within this industry, different products corresponding to each of the types can be developed by establishing seven categories, “Seven Dark Suppliers”, that could satisfy tourists’ motivations.

- Dark Fun Factories. This refers to places to visit and attractions that are centered on entertainment and commercial values where events related to death or the macabre are real or fictitious.
- Dark Exhibitions. This type offers products related to death and the macabre but with an air of commemoration and education. It is not simply fun or enjoyment, as in funfair factories; in this case, it is intended to teach something.
- Dark Dungeons. This refers to attractions that show both old prisons and old justice palaces.
- Dark Resting Places. This typology alludes to cemeteries as resting places and considers them as a potential product within “dark tourism”. It proposes using cemeteries as a mechanism to promote visiting areas and conserving the landscape and architecture, in addition to considering these locations as memorials.
- Dark Shrines. These are places where remembrance and respect is paid to a recently deceased person.
- Dark Conflict Sites. These are the activities, places and destinations that have a military conflict as their main motive. They have educational features but are, above all, commemorative and were at first not intentionally related to the concept of “dark tourism”.
- Dark Camps of Genocide. Concentration camps represent the places where atrocities and genocides have taken place.

Therefore, a tourist who participates in “dark tourism” can have different reasons for doing so and plan their trip according to their own wants. Europe, as an old continent, has many places to engage in dark tourism. In this research, we will focus on cemetery tourism in southern Europe, specifically in Andalusia.

## 3. Thanatourism: The European Cemeteries Route and Spain’s Unique Cemeteries

Called “necrotourism,” or cemetery tourism, this practice is a subsegment of dark tourism that is becoming increasingly important. Necrotourism is where tourists can walk the corridors of graveyards to discover the artistic, architectural, historical and scenic heritage treasured by cemeteries [31]. In the United States, the following cemeteries are noteworthy: the Green-Wood Cemetery in Brooklyn, New York, where guided visits by local historians are conducted, and Arlington National Cemetery, which is doubtlessly the most famous military cemetery in the world. It was founded during the American Civil War, and highlights include the eternal flame on the grave of John F. Kennedy. Mount Auburn Cemetery in Massachusetts is one of the most beautiful “parks” in the United States and not only contains the tombs of numerous famous people such as Henry Wadsworth Longfellow and Henry Cabot Lodge, which attract tourists, but is also populated by a variety of plants and birds. In Argentina, the Cementerio de la Recoleta in Buenos Aires, attracts visitors for its art embodied in sculptures and its stories.

In Europe, London’s Highgate Cemetery stands out as a cemetery of the Victorian era; today it is a natural reserve, with flowery pantheons and no lack of fallen or half-fallen angels and late-Gothic imagery. Zentralfriedhof in Vienna is where some of the most important musicians of all time rest: Beethoven, Schubert, Strauss, Brahms. This cemetery also contains the monuments to the fallen of the First World War and the country’s War of Independence. Père-Lachaise in Paris is one of the most visited cemeteries in the world [32], undoubtedly thanks to the number of famous people who rest in it, such as Oscar Wilde, Balzac, Delacroix, Chopin and Jim Morrison. The Jewish Cemetery of Prague is embedded among the centuries-old houses of the Jewish Quarter in Prague and is noted for the agglomeration of its tombs. According to the city’s archives, 100,000 people may be buried there, spread over 12,000 gravestones, data that speak to the little space that Prague granted to the Jewish ghetto at the time.

In Spain, cemeteries are valued; actions to preserve this heritage include the registration of approximately 20 Spanish cemeteries as points of cultural interest (bienes de interés cultural—BIC) or autonomous and/or local recognition (Goods of cultural interest (bienes de interés cultural—BIC) are understood as those that are registered and catalogued as such, while the recognition refers to those legal figures that lead to certain protection, such as the figure of cultural good of local interest of municipal scope). In recent years, cultural tourism has emerged as one of the main elements that stimulates heritage, and cemetery tourism is a part of this trend; therefore, cultural tourism favors the protection of cultural itineraries, i.e., conservation and valuation of both roads and the landscapes that accompany them. A cultural tourism itinerary highlights the monumental and historical-artistic aspect of the cemeteries, defining them as “open museums”, “open air museums”, “microcosms” or “local heritage sites”, and they are presented as an alternative way of visiting and getting to know cities and their history and traditions.

Since 1987, The Council of Europe has recognized 32 European Cultural Routes, of which 20 have part of their route in Spain. Among these is the European Cemeteries Route, which became part of the list in 2010 thanks to the momentum of the Association of Significant Cemeteries of Europe (ASCE). This route integrates 63 cemeteries in 56 cities in 20 European countries. The European Cemeteries Route has the presentation of European funerary heritage, tracing a polyphonic image in movement of customs, traditions and funeral art representative of the European continent in the last two centuries and presenting a vision of recent history as its fundamental objective. However, it also seeks to promote quality cultural tourism through the supply of new spaces while establishing transnational cooperation links, promoting “the restoration of the funerary landscape, converting these spaces into a tool of knowledge and continuous research and function with a clear educational vocation so that schools incorporate it into their cultural visits in a dynamic and pedagogical way.” As a tourist product, the cemeteries route represents an alternative and/or a complement to the existing offer.

Cemeteries are sacred and emotional spaces, but at the same time, they are witnesses to the local history of cities and towns. They are common to all cities and towns in Europe and therefore clearly reveal the cultural and religious identity of the city, forming part of the tangible heritage through their works, sculptures, engravings and even their urban planning. In the same way, cemeteries are part of the intangible heritage of our anthropological reality, supporting the environment that surrounds the habits and practices related to death and providing unique scenarios where we can find part of our historical memories. They are places to remember periods of local history that communities neither want to nor should forget and history that we have a duty to preserve and transmit to future generations, according to Dancausa et al. [32].

This route refers to cemeteries as places of life and environments that, as urban spaces, are directly linked to the history and culture of the community to which they belong and where we will find many of our references. The importance of the European Cemeteries Route lies in its multicultural diversity, which occurs mainly through the interaction between its members more than the simple value of its individual components. In Spain, the most unique cemeteries are found in the regions of Andalusia, Catalonia, Cantabria, Valencia and Asturias.

## 4. Methodology

In the treatment of information, a field study was carried out aimed at the population of tourists who visited a cemetery or “haunted” location in the province of Córdoba in 2019 (Table 1). To determine a profile and motivations for this, a questionnaire consisting of 24 questions divided into four sections was conducted.

1. The first block of the questionnaire collected personal information (age, gender, educational level, marital status, etc.);
2. The second block gathered information about the visit (Number of people who have come with you to complete the route, Has the visit met your expectations regarding the dark tourism route taken? Is the price paid in accordance with the route? How did you find out about the route?);
3. The third block questions about motivation to visit (How do you assess the current situation in terms of tourism management of places such as those you have visited? What do you think about the creation of a dark tourism route in the capital or in the province?);
4. The fourth block collected information questions about dark tourism locations regarding value (From your point of view as a customer, what is the greatest barrier to the development of sites for dark tourism? Do you know that there are similar sites to those visited in the province?).

Information was collected using a questionnaire directed at the population of tourist consumers visiting a cemetery or “haunted” location in the province of Córdoba. The access by the surveyors to the dark route (cemeteries, ghosts’ enclaves) and the conduct of interviews with tourists was authorized by the managing body and owner of the cemeteries.

Prior to the completion of the questionnaire, tourists were informed of academic purposes and anonymity in answering. Consent to take the questionnaire was verbal. At all times, the visitor’s anonymity to the dark route was guaranteed.

With both qualitative and quantitative information extracted from the questionnaire, a univariate descriptive analysis was performed to determine the percentage of each category of the variable (percentage by gender, age, income level, etc.) and a bivariate analysis was performed through contingency tables to analyze whether there is association or independence between two variables using the χ2 statistic.

The initial population (tourists) was complex, and the distribution of the sample was a function of the tourists who visited the four selected enclaves (Faculty of Law and Business Administration, Faculty of Philosophy and Letters, Palace of Orive and Cemetery of Our Lady of Health). With the objective of analyzing the variables that influence visit satisfaction, a logit model has been developed that explained the probability of being satisfied (Satisfaction) with the visit made to dark tourism places in Córdoba (dichotomous variable that takes the value of 1 if their satisfaction level with the visit is greater than 75% and 0 if it is not) with the aim of identifying the factors that influence satisfaction depending on the tourist’s profile.

Of all of the predetermined variables, the only ones that are significant to explain the probability of being satisfied with the visit are as follows:

Age (Age);
Family income (income);
Academic level, which was divided into three variables to highlight the main categories: university education (undergraduate, master, doctorate) (uni), intermediate education (baccalaureate and vocational training) (ba), and not completed an education or has a basic high school education (ncs), the latter being the reference variable.

An autoregressive integrated moving average (ARIMA) model [33,34], was also created to predict the demand for dark tourism in Córdoba, a province located in southern Spain. According to Gujarati [35], the facilitating factor of this prediction method is in an analysis of the probabilistic, or stochastic, properties of the economic time series (in this case, the number of dark tourists in Cordoba). In the time series models, the dark tourist variable can be explained over time by its past or lag values and by the stochastic error terms, giving ARIMA models an advantage of being less costly in data collection, as only historical observations of the data are required. In contrast, the main limitation of using univariate analysis is that it does not recognize any causal relationship with the behaviour of other endogenous variables or information related to the behaviour of other explanatory variables. The SARIMA models (p,d,q) × (P,D,Q)s are described by the following expressions:

$$\varphi \;\left(B\right)\;\Phi \left(B^{s}\right)\;Z_{t}\;=\;\theta \;\left(B\right)\;\Theta \left(B^{s}\right)\;a_{t}\;$$

$$Z_{t\;}={\left(1-B\right)}^{d}\;{\left(1-\;B^{s}\right)}^{D}\;Y^{\left(\lambda \right)}$$

where the operators introduced in the formulas are *Y_t_* (series observed, in our case, it is the gastronomic tourism demand, *λ* (represents the correction of the trend in variance of the series), *Z_t_* (series that is de-seasonalized and without a trend, that is, is stationary), *B* (lag operator), (1 − *B*) (typical difference operator), B^s^ (seasonal lag operator, (1 − *B^s^*): seasonal difference operator). The difference operators and seasonal difference operators, in general, eliminate the trends and the seasonal components of the series, respectively. ϕ(B) is the autoregressive polynomial of order *p*, corresponding to the ordinary part of the series; θ(B) is the polynomial of moving averages of order q, corresponding to the ordinary part of the series; Φ(B^s^) is the p-order autoregressive polynomial, corresponding to the seasonal part of the series; Θ(B^s^) is the polynomial of moving averages of order Q, corresponding to the seasonal part of the series; at is the disturbance of the model; and *D* is the number of times the seasonal difference operators and typical difference are applied to the original series to make it stationary.

In the ARIMA models, the behavior of a time series is explained from the past observations of the series itself and from the past forecasting errors. Several studies have shown how ARIMA models and their different variants obtain good results in forecasting tourism demand.

## 5. Results

The results obtained can be divided into three groups according to the techniques used:

### 5.1. Univariate and Bivariate Analysis

A descriptive analysis of the results shows that the profile of people who participate in dark tourism on the Andalusian cemeteries route is between 26 and 40 years old (59.4%) and with a university education (48.7%). These results are similar to those obtained by Carrión [33] in a study of the Yungay Cemetery in Peru. They are also single people (51.5%), mainly women (55.4%), who are attracted to the paranormal phenomena related to ghosts and want to know about tombs and everything related to the death of illustrious people buried in the cemeteries of Córdoba [34,35]. As indicated by Korstanje [36], the sites of dark tourism function as instructive devices about trauma, which evokes mass death in conditions of vulnerability of others (Table 2).

Also noteworthy is the 48.7% of these tourists who have a college education; therefore, they are visitors with a high level of education, differing from the profile of other tourists such as those at the Museum of the Mummies of Guanajuato (Mexico), wherein superstition plays a fundamental role, which receives more than 600,000 tourists per year with an average educational level of primary or secondary education.

People who visit dark tourism sites in Córdoba mostly come from the Autonomous Community of Andalusia (55.2%), demonstrating that this type of tourism can be further commercialized in the national and international markets. The Córdoba Cemeteries Route still attracts little foreign tourism (0.4%), far different from the profile of other cemeteries such as the Jewish Cemetery in Prague, where the percentage of foreign tourists exceeds 80%.

It has been observed that 68.1% of tourists would be willing to repeat a route with an experience similar to the one they had, and a degree of satisfaction greater than 76% allows for the creation of a commercial tourism product with high fidelity, since 91.6% of respondents rated their experience very positively.

Table 3 shows that this type of tourism is mainly discovered through the internet or social networks (53.8%), though the dark tourism web still has a low percentage of attraction compared to the demand for tourist information (13.5%). Additionally, it can be seen that dark tourism is not highly publicized in brochures or by tour operators; this behavior is similar to that observed in Malaysian research by Mohd et al. [37], who indicate that dark tourism, as a new tourist product, has a very low promotional effort made by tour operators until the demand increases.

If we compare the dark tourist in Córdoba with the profile of the cultural tourist in Spain from Soro et al. [38], it is clear they are different, although both have higher education levels; the age of the dark tourist is young, between 26 and 40 years, while the most common cultural tourist is between 46 and 55 years with a higher income level than dark tourists in Córdoba. Dark tourists also tend to travel as a couple versus cultural tourists, who tend to travel with friends and family.

Regarding tourists’ expectations about the route taken, 93.2% felt their expectations were met, indicating that the product offered is quality; however, this number could be improved by increasing audiovisual media (35.2%) or providing brochures explaining the visit (25.2%).

It should be noted that 55.2% of the tourists who took the route (either the ghosts, tourism or cemetery trips) traveled from their place of origin to take this tour; 88.1% considered the price appropriate when considering the product’s quality.

The main motivation dark tourists have for participating in Córdoba is the constant search for new paranormal experiences (74.3%), one of the main motivations described by Carrasco et al. [39], since people need to escape from daily monotony [40]. (Table 4).

Table 5 shows the knowledge and opinions of tourists regarding dark tourism routes in Córdoba; 86.4% know that there are similar sites of dark tourism that differ from those in Córdoba, and 77.7% have visited them. These tourists also believe that dark tourism is a good reason to visit Córdoba and the province. This percentage so high because respondents include only dark tourists. According to the tourism observations in Córdoba in 2016, the main reasons for general tourists visiting the city was to experience its material heritage and then its gastronomy.

Regarding the assessment of the tourism management of the places visited, 60.4% rated the management as good, and 93.2% thought that the route of cemeteries in Córdoba should be offered along with the route of ghosts or legendary places.

Based on the results of the univariate analysis, we can highlight that the dark tourist in Córdoba and the surrounding province is very satisfied with the tourism product, is knowledgeable about the topic and believes that combined products could be created; this tourist also appreciates the lack of coordination in tourism product management, partly due to the experience of having visited similar sites. Therefore, Córdoba has great market potential for this tourist niche, but adequate management and coordination between public and private entities is necessary to offer a quality product and increase the number of tourism offerings, especially at night. This will increase the number of overnight stays and the average spending of tourists, two handicaps that have been difficult to solve in Córdoba as these actions are often performed individually and not coordinated (Table 6).

With the aim of deepening the analysis between the different variables, a bivariate analysis was performed. There is a strong relationship between the tourist’s age and their degree of satisfaction with the route of cemeteries (χ2 = 34.3, *p* = 0.00). When the tourist is younger, he rates the route more positively. Age also influences how tourists discover the route (χ2 = 27.5, *p* = 0.00). Younger tourists use new technologies, the internet, social networks and tourism websites, while older tourists use recommendations from friends and family.

However, there is no relationship between the reason for the visit and age (χ2 = 0.24, *p* = 0.9997), gender (χ2 = 1.13, *p* = 0.5681) or income level (χ2 = 2.25, *p* = 0.9723).

It is also observed that there is a relationship between the variables if the tourist has visited other dark tourism sites and their degree of satisfaction; thus, people who have not visited other dark tourism sites are more satisfied than those who have seen other places, because they make a comparison, and although the tourism product in Córdoba is good, its youth and lack of coordination do not match those of other places (χ2 = 32.3, *p* = 0.00).

Additionally, level of education and degree of satisfaction are correlated. When the tourist has a higher level of education, their degree of satisfaction is somewhat lower, either because the historical explanations of the legendary sites are not very accurate or because they believe that audiovisual media are lacking. (χ2 = 25.1, *p* = 0.00).

A logit regression model (Table 7) has been performed to calculate the probability that a tourist is satisfied with the visit made to the dark tourism place (cemetery or places with ghosts) and is attached to the final model where the variables are significant at 5%).

From these estimate, we obtain the following results:

All of the variables of the best logit model obtained negatively influence the probability of being satisfied, with the most relevant variables being university academic level (−1.24). If the tourist has a university education, he/she is less satisfied with the visit than those who have not completed an education or have a basic education, corroborating the results of the contingency table.

The level of income has a negative influence; as the visitor’s income increases, his/her satisfaction with the visit decreases. Tourists with higher income levels are generally those who have a university education.

The age variable negatively influences the probability of being satisfied; older tourists have lower degrees of satisfaction.

It should be noted that there are no significant differences between males’ or females’ probability of being satisfied, and this variable is not significant.

Therefore, cemetery and ghost tourism is a niche market that is not sufficiently exploited in Andalusia and, thus, has significant potential for development. When combined with other forms of dark tourism, such tourism could generate wealth and expand the cultural offerings in Andalusia and, specifically, in Córdoba.

### 5.2. Arima Model for Forecasting Demand for Dark Tourism

Dark tourism in Córdoba is a tourist segment little exploited due to its novelty but growing very slowly. To make it more dynamic, studies indicate evolution is needed, as are studies that apply the necessary marketing tools to make this tourism more dynamic and turn it into an attractive product for the cemeteries at the location has and places of legend.

There are no studies on the potential demand for dark tourism in Córdoba, and this article is a novel contribution that covers this research gap. This absence of studies is mainly due to the difficulty of obtaining data on tourists, since the statistics held by companies dedicated to tourism are scarce. In this research, we have compiled the information from tourists who carried out different dark tourism routes. The majority of the tourists of this typology are concentrated using ARIMA models, widely used for the prediction of tourists [41,42,43,44,45,46,47,48,49,50].

**Figure 1:**
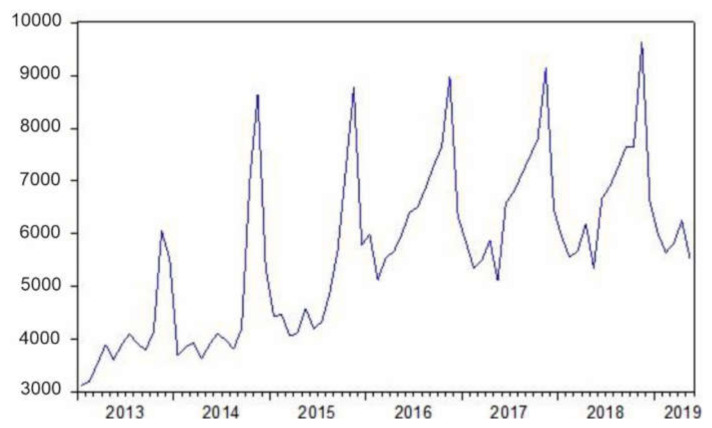
Evolution of the demand for dark tourism in Córdoba (January 2013–May 2019). Source. By authors.

Figure 1 shows a slight upward trend in the demand variable over the seven years analyzed (January 2013 to May 2019). It can also be seen that this variable has a tendency in variance, which has been corrected with the transformation of Box-Cox λ = 0.3, and a tendency in mean and in cycle that have been corrected with a differentiation in mean and cycle.

The estimated monthly forecast model of demand for dark tourism being seasonal ARIMA (2,1,0) (1,1,0) 12 (Table 8)

$$\left(1+0.089857B^{2}\right)\left(1-0.099307B^{12}\right)\;{\left(1-B\right)}^{1}\;{\left(1-B^{12}\right)}^{1}Tourists^{0.2}=\;a_{t}$$

$$t_{\varphi 2}=\;-6.30199^{*}\;\;\;\;\;\;\;\;t_{\phi 1}=\;-9.88241^{*}$$

*Significant parameters for α = 0.05

Table 9 and Table 10 show the different validation contrasts of the model, such as the Ljung-Box test, and verify that the null hypothesis of absence of autocorrelation is met, since the statistical probability is higher than the 1% significance level of the model (“Prob” column).

The autoregressive conditional heteroskedasticity (ARCH) statistic (Table 10) indicates that in the model, there is no conditional autoregression heteroskedasticity (null hypothesis), since the probability of a statistic 0.2045 above the level is 0.05.

Table 11 shows the value of the extended Dickey Fuller statistic (−1.75), indicating that this value satisfies the null hypothesis of a unit root.

Table 12 shows that the month with the fewest visits is May (5730). This value is contradicted by the number of tourists that Córdoba receives, since May is the month with the greatest number of visitors by far, as it is when the Fiesta of the Patios (declared to be Intangible Cultural Heritage by UNESCO), the Wine Fest, the Crosses of May festival and the Fair of Our Lady of Health all take place. This finding indicates that the tourists that Córdoba receives are mainly day trippers and that they rarely stay overnight. Overnight stays and average spending are one of the weaknesses that should be solved by increasing complementary supply, especially nightlife; dark tourism fits in this segment, especially ghost activities, as it is mainly done at night. Dark tourism could be a good resource to encourage tourists to spend the night.

This figure also shows the comparison of the months of June to December of 2018 and January to May of 2019. With respect to the predictions obtained in the SARIMA model from June to December 2019 and from January to May 2010, we see that in all months, there is a slight increase compared to the previous year, which indicates that this type of tourism is in the take-off or introduction phase, as is the case for all new products entering the market. This finding indicates slow growth, which could have a greater impact if the Córdoba City Council allowed visits for more days and the carrying out of activities related to the world of death and extended the opening hours of buildings of public bodies into the evening. 

A coordinated route of the two legend-rich cemeteries of Córdoba on the European Cemeteries Route should also be prepared. Right now, no companies or travel agencies have such a program. Tours should also be paired with food and drink products related to the time of the dead and the typical gastronomy of the area, and programs in cemeteries that are not focused on only historic age of the dead but also the cemetery’s historic and heritage value should be conducted year-round, as this would avoid the great seasonality that this tourist segment has.

## 6. Discussion

Dark tourism is strongly related to culture and heritage tourism having history and tragedies as a vital part of it. The places of dark tourism in Córdoba are mainly visited by people between 26–40 years old, the profile coinciding with Niela’s studies (House of Terror museum Budapest) [51].

Dark tourism is a special type of tourism business which doesn’t appeal to everyone, Visiting dark sites can induce both positive and negative experiences. Generally, the major attractions at dark tourism places raise negative emotional experiences like feelings of vengeance, fear, horror, depression, sadness, empathy etc. While tourists are in most cases motivated by a need for an educational experience, some ’have culture experience, coinciding with Stone’s research [52]. The main motivations for doing so in Cordoba are to know paranormal stories and visit cemeteries, they want to know tragic deaths and not so much the architectural heritage of the tombs (motivations similar to tourists visiting the Jewish cemetery of Prague) [13].

The study that was carried out showed that many places in Córdoba are feasible for implementing so-called dark tourism, especially ghost activities, and routes can be linked between municipalities, which allows for experiential tourism because paranormal experiences are very special occurrences that transport consumers to “otherworldly” destinations and make for unforgettable, unfamiliar, and supremely shareable memories and stories [53].

From this research it can be inferred that dark tourism in Cordoba has a very slight demand unlike other destinations of dark tourism as reflected in the studies of the Arlintong Cemetery in the USA [54,55] or Pére Lachaise Cemetery in Paris, being wasted this cultural offer.

## 7. Conclusions

Dark tourism in Córdoba continues to be a minor segment compared to other segments of cultural tourism, such as heritage or gastronomy tourism. To increase demand, it is essential to offer a product that meets the needs of the current consumer, so it is necessary to know their profile [47].

The routes of Dark Tourism in Córdoba are in the early stages of tourism management, with local tourism as the current market. Foreign tourism should be promoted, which would encourage spending the night in the city, increasing the average daily expenditure per tourist.

Regarding cemetery tourism, the cemeteries of Córdoba have a large number of funerary monuments that, due to their architectural beauty, landscape and botanical design, form part of the European Cemeteries Route. These unique Andalusian cemeteries, part of the route “Memories of Andalusia”, constitute a tourist attraction that is still to be exploited in Córdoba. This is clear because there is no possibility of night visits or ghost tours (or paranormal phenomena). 

Ghost tours should be explored because there are many houses in Córdoba that have some legend or ghost that lingers in them and are therefore an attraction that has even been analyzed in specific television and radio programs, this may represent a novel market niche that can generate wealth, not only from the tickets paid to dark tourism companies for tours of cemeteries or ghost locations but also for hotels, which would benefit because tourists would spend the night to attend these nocturnal tours. Bars and restaurants where tourists eat and souvenir shops where they purchase memories of the places they visit would also benefit. Houran et al. [48] estimated that paranormal tourism generates at least $100 million worldwide, with Córdoba and, thus, Spain being places where dark tourism can grow in the future.

From the sites analyzed, it can be deduced that dark tourism, especially that of cemeteries, is not configured as an attractive tourist product. A good strategic plan is needed to develop this tourism segment in a sustainable manner and become an all-season tourist draw for cultural tourism in the city, as is possible because of the high degree of satisfaction with the visit, similar to the studies of Moh et al. [37]. Uncertainty, charm, emotion, authenticity, and expectations of what may happen or be seen at a destination are variables that make certain places that carry the legend of Córdoba destinations unique and attractive to a special tourist [49]. 

Proper professionalization of the sector could make the cemeteries of Córdoba a reference point and draw thousands of foreign tourists, increasing this market share from almost zero in Córdoba (below 1%) to perhaps the level of other cemeteries such as the Prague Jewish Cemetery or Arlington in the U.S.

The tourist profile of dark tourism participants in Córdoba is young, has a university education, seeks new experiences, is aware of dark tourism places through the Internet, shares positive and negative opinions of and experiences at those sites through the “electronic word of mouth” (eWOM) network [50,51,56,57,58], is very satisfied with the dark type of tourism, and would repeat the experience by visiting other cemetery routes. Therefore, this customer is a loyal tourist who is willing to pay for a quality product.

Therefore, more studies are needed to analyze supply and demand, as one of the main difficulties encountered is the lack of information on this type of tourism broken down at the monthly level over several years.

As seen when using the ARIMA model, there is a potential growing seasonal demand for celebrations of the dead, and this type of tourist is satisfied. However, the existing supply in the city and province should be made known. For such an effort, all of the agents involved must unite in order to match the results obtained in other European cemeteries; all agents should also promote and help old houses because rehabilitating them is very costly and they are emblematic houses of the city that vulnerable to destruction, losing the legends that accompany them.

Dark Tourism can be an element that complements the nocturnal leisure activities that make the city of Córdoba a place to spend the night and provide education about the city’s heritage and the legends of different cultures that have formed a paranormal spectrum that can generate economic activity in the city.

Dark tourism is a new market niche with great potential in Córdoba that can be extrapolated to the rest of Spain because cities such as Toledo, Granada, and Barcelona are places where paranormal phenomena and violent deaths have occurred over the centuries, creating legends among the population. Through a good advertising campaign and by promoting dark tourism websites, these places could attract tourists who desire to have experiences of terror or death, thus satisfying their need to have macabre experiences.

This research has potential limitations, the first of which is the sample used. The data was only obtained from dark tourists in Cordoba, which could indicate that the collected data is only applicable to one unique tourist site. Another limitation of this studio is the ARIMA model. It was applied for lawsuits prior to the devastating effect of COVID-19 on tourism. Therefore, the demand forecast will be valid for when past tourist flows are re-established. Future lines of research could include other internal and external variables, as well as studying of the relationships between destiny, motivations and satisfaction.

## Figures and Tables

**Table 1:** Datasheet of the survey.

Survey Characteristics	Offered Survey
Population	People over the age of 16 who have carried out dark tourism (Cemeteries and ghosts) in Córdoba and Province
Sample size	223 valid surveys
Sample error	+/− 4.05%
Level of confidence	95% *p* = q = 0,5
Fieldwork dates	January 2019–December 2019

Source: By authors.

**Table 2:** Profile of the tourist of dark tourism of Córdoba and Province.

Block	Question	Classification	Percentage
D. Questions related to the characteristics of the dark tourist	Age range	16 years to 17 years	0.9%
		18 years to 25 years	17.3%
		**26 years to 40 years**	**59.4%**
		Older than 40 years	22.4%
	Level of education	No completed studies	0.6%
		High school	17.3%
		Baccalaureate	33.4%
		**University**	**48.7%**
	Gender	Man	44.6%
		**Woman**	**55.4%**
	Marital status	**Single**	**51.5%**
		Married	40.1%
		Divorced/separated	7.2%
		Other status	1.3%
	Monthly income level of the family unit	Less than 1000 euros	29.3%
		**1001–1500 euros**	**39.4%**
		1501–2000 euros	10.6%
		2001–2500 euros	9.3%
		More than 2500 euros	11.4%
	With whom did you experience the tour?	Alone	3.6%
		**Accompanied by my partner**	**43.1%**
		With friends	37.4%
		With family	15.9%
	What is your origin?	Cordovan	40.3%
		**Rest of Andalusia (except Córdoba)**	**55.2%**
		Other Autonomous Communities (except Andalusia)	4.1%
		Abroad	0.4%
	Would you repeat the experience with a similar route?	**Yes**	**68.1%**
		No	31.9%
	Satisfaction level of the visit	Less than 25%	1.6%
		25–50%	2.1%
		51–75%	4.7%
		76–99%	36.2%
		**100%**	**55.4%**

Source: By authors. (bold the largest percentages of variable).

**Table 3:** Univariate results of the survey of people who participated in dark tourism. in Córdoba: Questions about the visit.

Block	Question	Classification	Percentage
A. Questions about the visit	Number of people who have come with you to complete the route	1 person	3.5%
		**2 to 4 people**	**72.1%**
		More than 4 people	24.4%
	Has the visit met your expectations regarding the dark tourism route taken?	**Yes**	**93.2%**
		No	6.8%
	What would you improve?	Nothing	20.3%
		**More audiovisual media**	**35.1%**
		Delivery of written material on the route	25.2%
		Historical data	9.3%
		Other	10.1%
	Would you be interested in receiving more information after your visit?	**Yes, if it is free**	**52.1%**
		Yes, in any case	28.3%
		I do not consider it necessary	19.6%
	Did you come specifically to carry out this route, or was it offered to you in Córdoba?	**I came expressly from my place of origin**	**55.2%**
		It was circumstantial; they offered it to me	44.8%
	Is the price paid in accordance with the route?	**Yes**	**88.1%**
		No	11.9%
	How did you find out about the route?	Through the internet, on dark tourism websites	13.5%
		**Through the internet, on social networks**	**53.8%**
		From the recommendation of friends and family	22.3%
		Through printed brochures	3.6%
		Other means	6.8%

Source: By authors. (bold the largest percentages of variable).

**Table 4:** Univariate results of a survey of people who carried out dark tourism in Córdoba: Motivations.

Block	Question	Classification	Percentage
C. Questions about motivation to visit	What most motivates you to visit?	**To experience paranormal stories**	**74.3%**
		Visit a cemetery to see its tombs, sculptures	23.1%
		See sites where battles have been held	2.9%
	How do you assess the current situation in terms of tourism management of places such as those you have visited?	**Good**	**64.1%**
		Average	30.3%
		Bad	5.6%
	What do you think about the creation of a dark tourism route in the capital or in the province?	**I agree**	**97.2%**
		I do not agree; I prefer to visit one site and not several	2.8%

Source: By authors. (bold the largest percentages of variable).

**Table 5:** Univariate results of the survey of dark tourists in Córdoba: Questions about dark tourism locations.

Block	Question	Classification	Percentage
B. Questions about dark tourism locations	Do you know that there are similar sites to those visited in the province?	**Yes**	**86.4%**
		No	13.6%
	Have you undertaken other dark tourism routes other than Córdoba?	Yes	22.3%
		**No**	**77.7%**
	Do you think dark tourism is a good selling point to visit Córdoba and the province?	**Yes**	**88.3%**
		No	7.1%
		I do not know; I cannot comment	4.6%
	Would you carry out a combined route of Córdoba cemeteries and ghosts?	**Yes**	**93.2%**
		No	6.8%
	From your point of view as a customer, what is the greatest barrier to the development of sites for dark tourism?	Discoordination of actions between different public administrations	13.6%
		**Disinterest of public bodies; they are not interested in dark tourism**	**60.4%**
		Lack of local private initiative; there are no entrepreneurs who invest in this area	23.1%
		Other (indicate)	2.9%

Source. By authors. (bold the largest percentages of variable).

**Table 6:** Results of the bivariate analysis of the demand for dark tourism in Córdoba.

Associated Variables	χ^2^	Df	*p*-Value
Age of the tourist/degree of satisfaction with the dark tourism route completed	34.3	12	0.00060
Degree of satisfaction with the dark tourism route carried out/cultural level	32.3	4	0.00001
Age of the tourist/knowledge of the route	27.5	15	0.02491
Degree of satisfaction with the dark tourism route completed/cultural level	25.1	12	0.00004

χ2 Chi-square statistic. Correlated variables for α = 0.05, df = degrees of freedom. Source: By authors.

**Table 7:** Estimation logit model.

Dependent Variable: SATISFACTION				
Method: ML—Binary Logit (Quadratic Hill Climbing)				
Included Observations: 123				
Variable	Coefficient	Std. Error	z-Statistic	Prob.
C	3.393223	0.975454	3.478611	0.0005
EDAD	−0.035456	0.016227	−2.085000	0.0376
INCOME	−0.000355	0.000109	−3.256885	0.0012
UNI	−1.248397	0.556890	−2.241729	0.0250
BA	−0.437861	0.215638	−2.030537	0.0424
McFadden Rsquared	0.426777	Mean dependent var		0.707317

Source: By authors.

**Table 8:** Estimation of demand for dark tourism in Córdoba SARIMA (2,1,0) (1,1,0)_12_. Dependent Variable: D(TOURISTS^0.2,1,12). Sample (adjusted): 2015M04 2019M05

Variable	Coefficient	Std. Error	t-Statistic	Prob.
AR(2)	−0.898567	0.142585	−6.30199	0.00001
SAR(12)	−0.099307	0.010048	−9.88241	0.00000

Source: By authors.

**Table 9:** Statistical analysis of Ljung-Box.

Autocorrelation	Partial Correlation	r	AC	PAC	Q-Stat	Prob
**| . |	**| . |	1	−0.293	−0.293	4.5398	
| . |	| . |	2	0.027	−0.064	4.5788	
*| . |	*| . |	3	−0.134	−0.159	5.5777	0.018
|*. |	| . |	4	0.136	0.056	6.6285	0.036
| . |	| . |	5	−0.028	0.024	6.6722	0.083
| . |	| . |	6	−0.007	−0.017	6.6754	0.154
*| . |	| . |	7	−0.075	−0.065	7.0113	0.220
| . |	| . |	8	0.011	−0.046	7.0186	0.319
*| . |	*| . |	9	−0.146	−0.190	8.3657	0.301
| . |	*| . |	10	−0.017	−0.154	8.3843	0.397
|*. |	| . |	11	0.100	0.048	9.0544	0.432
| . |	|*. |	12	0.065	0.091	9.3452	0.500
| . |	|*. |	13	0.016	0.105	9.3626	0.588
**| . |	*| . |	14	−0.212	−0.173	12.620	0.397
|** |	|*. |	15	0.230	0.115	16.553	0.221
**| . |	**| . |	16	−0.210	−0.214	19.938	0.132
|*. |	| . |	17	0.156	−0.013	21.853	0.112
*| . |	*| . |	18	−0.160	−0.105	23.927	0.091
|*. |	| . |	19	0.154	0.061	25.924	0.076
*| . |	| . |	20	−0.143	−0.029	27.686	0.067
| . |	*| . |	21	−0.015	−0.077	27.706	0.089
| . |	| . |	22	−0.004	−0.001	27.708	0.117
|*. |	| . |	23	0.148	0.017	29.814	0.096
*| . |	| . |	24	−0.088	−0.038	30.586	0.105

* The * represent the values of the autocorrelation functions (AC) and Partial correlation.

**Table 10:** ARCH test of heteroskedasticity.

Heteroskedasticity Test: ARCH			
F-statistic	1.655546	Prob. F(1,47)	0.2045
Obs*R-squared	1.667266	Prob. Chi-Square(1)	0.1966

Source: by authors. * Indicates multiplication.

**Table 11:** Dickey Fuller Test

Null Hypothesis: TOURISTS Has a Unit Root				Prob.
Exogenous: Constant				
Lag Length: 11 (Automatic—based on SIC, maxlag = 11)				
Test			t-Statistic	Prob.*
Augmented Dickey-Fuller test statistic			−1.750661	0.4014
Test critical values:	1% level		−3.534868	
	5% level		−2.906923	
	10% level		−2.591006	

Source: by authors; * MacKinnon (1996) one-sided *p*-values.

**Table 12:** Tourism demand predictions (Increase).

Month	June 2018–May 2019	June 2019–May 2020	Difference
June	6.665	6.921	256
July	6.897	7.144	247
August	7.245	7.499	254
September	7.634	7.896	262
October	7.645	7.940	295
November	9.645	9.929	284
December	6.623	6.855	232
January	6.003	6.227	224
February	5.643	5.825	182
March	5.821	6.032	211
April	6.246	6.479	233
May	5.534	5.730	196
Total	**81.601**	**84.477**	**2.876**

Source: by authors.

## Data Availability

The data presented in this study are available on request from the corresponding author.
